# Editorial: Microenvironmental stimuli-responsive nanomedicine for biomedical application

**DOI:** 10.3389/fbioe.2023.1206895

**Published:** 2023-05-04

**Authors:** Donglin Xia, Jingwei Xie, Yong Hu

**Affiliations:** ^1^ College of Engineering and Applied Sciences, Nanjing University, Nanjing, China; ^2^ School of Public Health, Nantong University, Nantong, China; ^3^ Department of Surgery-Transplant College of Medicine, University of Nebraska Medical Center, Omaha, NE, United States

**Keywords:** nanomedcine, stimuli-reponsive, nanoparticles, tumor, targeting

The purpose of this Research Topic, *Microenvironmental stimuli-responsive nanomedicine for biomedical application*, is to bring together the latest developments from researchers working on smart nanomaterials for biosensing and therapy applications. The guest editorial team would like to thank all colleagues who submitted their reviews and research articles for the Research Topic.

The releasing of a microenvironment-responsive drug in the morbid site is one of the most effective therapeutic approaches, especially nanoparticles, for enhanced therapeutic outcomes for tumor therapy. That is because the compromised potency of nanomedicines has been attributed to its limited delivery efficiency into tumors, with less than ∼1% of the nanoparticle dose reaching the solid tumors. Shen et al. analyzed the clinical value of magnetic resonance-guided microwave ablation in lung cancer. It showed that MRI-guided percutaneous ablation had significant prospects for the treatment of lung tumors and provided a satisfactory outcome. This suggested that local drug delivery could achieve favorable therapeutic efficacy. As it could not only significantly increase the local drug concentration but also decreased the number of drug administrations, it improved compliance and minimized side effects.

Stimuli-responsive drug delivery systems are promising for the control of drug release *in vivo*. Various responsive systems triggered by microenvironment stimuli have been widely reported in the literature for controlled drug release studies. Among all types of stimuli-responsive drug delivery systems, pH-sensitive releasing has received increasing attention. Unlike the direct response to pH, Yang et al. developed an indirect pH-responsive insulin release system, which regulated insulin release behavior for diabetes therapy. In this work, glucose oxidase was employed as the microenvironment-responsive switch and converted a change in the hyperglycemic environment to a pH-stimulus to control the insulin releasing behavior. Furthermore, the regulation of the local microenvironment by ultrasound altered the release behavior of the insulin, because ultrasound can generate reactive oxygen species (ROS) and regulate the pharmacological effects in a timely manner. Inspired by this, Chen et al. showed a remote ultrasound-induced lidocaine delivery system for postoperative pain management. Under remote stimulation, drugs were released into the bloodstream because of the high-concentration ROS microenvironment. These results suggest an effective strategy to spatiotemporally control the release of drugs.

As increasing attention has been paid to the treatment of tumors, research in microenvironmental stimuli-responsive nanomedicine has become active, and a wide range of work has been executed to enhance tumor treatment efficacy. Yang et al. reviewed the recent advances in potentiating the oxygenation in tumor tissue with nanomaterials and highlighted the superiority of microenvironmental stimuli-responsive nanomaterials in enhancing the therapeutic effect in tumor treatment. In order to strengthen the mitochondrial respiration suppression efficacy of atovaquone, Li et al. developed a targeting strategy with RGD-modified silk fibroin-based nanocarriers. An increased inhibition efficacy, enhanced chemotherapy effect, and strikingly suppressed tumor development was observed in the tumor models treated by the RGD-modified silk fibroin-based nanocarriers due to the targeting ability of RGD. These results suggest that an RGD-based targeted drug carrier could reverse the hypoxia microenvironment *in vivo* for enhancing chemotherapy, thereby suggesting a promising candidate for tumor therapy. Moreover, therapeutic specificity might also be achieved by targeting tumor cell-specific metabolic alterations. Previous studies suggested that most of the cancer cells exhibited the phenomenon of glutamine (GL) addiction, that is, the tumor cells actively absorb and accumulate GL in tumor tissues for growth. Zhang et al. showed an oxygen-deficient TiO_2-x_ coated with a GL layer for targeted delivery with the joint application of sonodynamic therapy and photothermal therapy. This study presented a nanomedicine with high target efficacy to the tumor.

The studies about microenvironmental stimuli-responsive nanomedicine for biomedical application are not limited to abovementioned research. Recently, Zhang et al. summarized the mechanism of action, administration methods, and engineered production for exosomes in medical aesthetics. However, exosomes used as drug delivery vehicles still face many challenges in clinical practice. Shi et al.studied the nasopharyngeal carcinoma immune microenvironment through ferroptosis-related genes. The results show that ATG5 has potential as a significant independent prognostic marker and might be used to attain drug targeting. Zhao et al. studied the role of Notch signaling and fluid shear stress in regulating osteogenic differentiation. The results reveal new information concerning the osteogenic differentiation of hMSCs under shear stress and the regulatory role of Notch signaling. Enhancing targeting efficiencies for drug delivery applications could be pursued by considering those findings as microenvironmental stimuli-responsive candidate switches.

In summary, this Research Topic has collected diverse aspects of microenvironmental stimuli-responsive nanomedicine for biomedical applications. More specifically, it illustrates a variety of microenvironmental stimuli-responsive switches with different stimuli-responsive properties, and relevant stimuli-responsive mechanisms are systematically investigated and presented. The development of microenvironmental stimuli-responsive nanomedicine is a fascinating subject, and the current Research Topic is anticipated to provide a valuable reference for the exploration of this hot research field.

